# Regional Comparison of Goblet Cell Number and Area in Exposed and Covered Dry Eyes and Their Correlation with Tear MUC5AC

**DOI:** 10.1038/s41598-020-59956-7

**Published:** 2020-02-19

**Authors:** Karima S. Khimani, Jonathan A. Go, Rodrigo Guimaraes De Souza, Travis Mitchell, Zhiyuan Yu, Cintia S. de Paiva, Meghan Saumur, Stephen C. Pflugfelder

**Affiliations:** Department of Ophthalmology, Baylor College of Medicine, Houston, TX United States

**Keywords:** Eye diseases, Biomarkers

## Abstract

To compare goblet cell (GC) number and area in the covered superior (SB) versus exposed temporal (TB) bulbar conjunctiva in control versus aqueous tear deficient eyes (ATD) and evaluate correlation with tear MUC5AC protein. SB and TB impression cytology performed on control eyes, Sjögren syndrome (SS) ATD, and non-SS ATD was stained with period acid Schiff. GC number and area were measured with image analysis software. Protein-normalized MUC5AC level was measured in Schirmer strip-collected tears. Compared to control conjunctiva, GC number and area were significantly lower in SS, non-SS, and combined ATD groups in exposed TB, and were also significantly lower in SS and combined ATD groups in covered SB. In all ATD, GC number and area were significantly correlated, but differences between SB and TB were non-significant. Normalized tear MUC5AC protein was lower in all ATD groups versus control eyes, and correlated only with GC area. GCs are significantly decreased in the covered and exposed conjunctiva in SS. GC area may be a better disease measure than number for ATD. Correlation between tear MUC5AC concentration and GC area suggests tear MUC5AC mucin can be used as a disease-relevant biomarker for conjunctiva GC health.

## Introduction

Conjunctival goblet cell products are essential for maintaining tear stability and ocular surface immune tolerance^1–3^. Studies utilizing conjunctival impression cytology, taken primarily from the exposed conjunctiva, have found a decrease in goblet cell number in aqueous tear deficiency (ATD) and ocular surface inflammatory diseases, such as Sjögren syndrome, Stevens Johnson Syndrome and graft-vs.-host disease (GVHD)^4–9^. The T cell cytokine interferon gamma (IFN-γ) causes secretory dysfunction and death of conjunctival goblet cells^10,11^. The density of lymphocytes is highest in the non-exposed superior conjunctiva in control eyes, and mature antigen presenting cells (APCs) that prime naïve T cells were found to be higher in this region compared to the exposed conjunctiva in the autoimmune disease Sjögren syndrome^12,13^. Additionally, goblet cells have been reported to be reduced or absent in the superior bulbar conjunctiva in superior limbic keratoconjunctivitis (SLK), a condition that is often accompanied by ATD^14^.

Impression cytology is a costly, time consuming test that is often only available at academic centers^15,16^. Methods to analyze goblet cell number and size in impression cytology are not standardized, and it has not been established if measurement of goblet cell number or area shows the greatest discrimination between control and dry eyes^17^. Standardized methodology to measure conjunctival goblet cells is needed before goblet cell measurements can be utilized as a clinical trial efficacy parameter. Additionally, there is need for a clinically relevant tear assay that correlates with goblet cell number/function^18^. The primary objective of this study was to compare goblet cell number and area in the non-exposed superior bulbar (SB) and exposed temporal bulbar (TB) conjunctiva to determine which site shows greater difference between control and dry eyes. A secondary objective is to determine if tear MUC5AC protein is reduced in ATD and correlates with goblet cell number or size in impression cytology and clinical severity markers.

## Methods

### Study populations

The study was conducted in accordance with the Declaration of Helsinki. The protocol and informed consent form were approved by the Baylor College of Medicine Institutional Review Board prior to study initiation. Written, informed consent was obtained from all participants after explanation of the purpose and possible consequences of the study. Aqueous deficient dry eye patients and control subjects were recruited from the Ocular Surface Disease clinic at Baylor College of Medicine. Patients with superior limbic keratoconjunctivitis (SLK) were excluded from this study.

A standardized ocular surface evaluation including Symptom Assessment Questionnaire in Dry Eye (SANDE)^19^, fluorescein tear break-up time (TBUT), Schirmer I test, cornea fluorescein and conjunctival lissamine green dye staining, and tear meniscus height (TMH) measured by optical coherence tomography (OCT) were performed, as previously described^5,20^. To measure TBUT, fluorescein was placed on the inferior tarsus via a fluorescein strip (BioGlo, HUB, Rancho Cucamonga, CA) wet with preservative-free saline. Following several blinks, the patient was observed under cobalt blue light while spontaneously blinking, and TBUT was defined as the time elapsed in seconds from the last blink to the appearance of the first break in the continuous layer of fluorescein. To quantify tear production, Schirmer I test without anesthesia was performed. A dry Schirmer test strip was placed over the outer one-third of the lower eyelid margin. Schirmer I score was defined as the distance that tears wet the test strip after 5 minutes. Corneal fluorescein staining was graded 0 to 3 in each of 5 zones (central, temporal, nasal, superior, and inferior) with a total maximum score of 15^21^. Conjunctival lissamine green staining was graded on a scale of 0 to 3 in the exposed nasal and temporal bulbar conjunctiva with a total maximum score of 6. The ocular surface clinical parameters were all measured by the same observer (S.C.P.). Diagnostic criteria for ATD included SANDE score > 40, TBUT ≤ 7, TMH < 250 μm and Schirmer 1 < 10 mm. Patients with SS met proposed American College of Rheumatology diagnostic criteria for SS^22^.

Control subjects had no eye irritation, a TBUT ≥ 8 seconds, Schirmer 1 ≥ 10 mm, tear meniscus height ≥ 250 μm, no corneal fluorescein staining, and no meibomian gland disease. Subjects were excluded if they had prior LASIK or corneal transplantation surgery, cataract surgery in the past year, punctal occlusion with plugs or cautery, a history of contact lens wear, use of topical medications other than preservative-free artificial tears, or chronic use of systemic medications known to reduce tear production. They were instructed not to instill any tear drops on the day of the evaluation.

### Conjunctival goblet cell number

Goblet cell number was measured in impression cytology specimens taken from the TB and SB conjunctiva of the left eye with the EyePRIM device (OPIA, Paris, France). Membranes were fixed and stained by periodic acid Schiff (PAS) reagent, as previously described^5^. Each specimen was examined under brightfield, light microscopy at 10x magnification and representative images of four different regions in each specimen were captured using a Nikon DS-Fi1 microscope camera. Camera settings were standardized at 1,280 × 960 pixels, with auto exposure, auto white adjustment, high contrast, and exposure compensation of +1.0 exposure value (EV).

The visible goblet cells in each specimen were measured with image analysis software (Nikon Elements, Garden City, NY, USA), set with a standard resolution of 2.43 um/pixel. With a standard image size of 1,280 × 960 pixels, the viewing area for each image was normalized at 3,110.4 um × 2,332.8 um.

**Figure 1:**
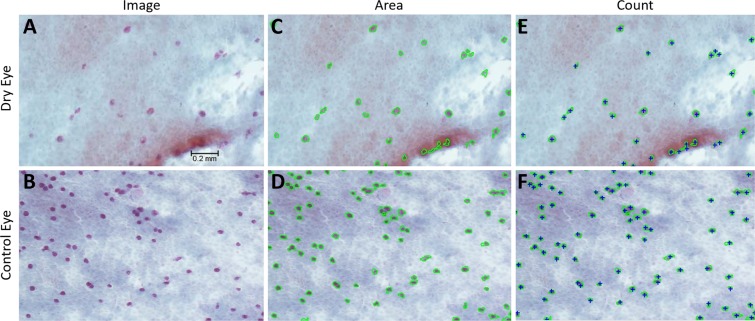
Images of impression cytology taken from the temporal bulbar and superior bulbar conjunctiva of the left dry (**1a**) and control (**1b**) eyes captured with a Nikon DS-Fi1 microscope camera under 10x brightfield, light microscopy. Nikon Elements software was utilized to measure the goblet cell area (**1c, 1d**) and goblet cell count (**1e, 1f**) of each specimen.

Goblet cells were identified by their PAS positive staining and circular appearance (Fig. 1a,b). For each specimen image, the areas of individual goblet cells were calculated with the software’s “Auto Detect” feature. After “Auto Detect” circumscribed goblet cells in green (Fig. 1c,d), the measurement was checked by a researcher, adjusted if needed, and saved as individual data points. The data points were exported to a spreadsheet program, and were summed by specimen image. The total goblet cell count was tallied for each specimen image using the software’s “Count” feature, which created blue “+” signs to mark each goblet cell (Fig. 1e,f). Total goblet cell area was expressed in mm^2^, and total goblet cell count was expressed as goblet cells/image. After a total goblet cell area and count was calculated for each specimen image, the four image measurements per original specimen were averaged and statistically analyzed.

### Tear MUC5AC ELISA

Unmarked Schirmer strips (Haag-Streit, Mason, OH) were used to collect tears. After measuring the length of strip wetting with tears, the non-wet portion was excised and the wet portion was placed in a screw top plastic tube and frozen at −80 °C. Defrosted strips were cut into small pieces, incubated in 30 μl 0.05% Tween 20 for one hour at 37 °C and centrifuged at 14,000 revolutions per minute (rpm) for 10 minutes at 4 °C. Protein concentrations were measured using a Micro BCA protein assay kit (Thermo Fisher Scientific, Waltham, MA; Cat# 23235).

Prior to ELISA, 2 μg protein per sample was treated with α2–3,6,8,9-Neuraminidase (Millipore, Burlington, MA; Cat# 480716) at 37 °C for two hours. The MUC5AC ELISA was performed following the manufacturer’s protocol (Cloud-Clone Corp, Katy, TX; Cat no. SEA756Mu). Absorbance measured at 450 nm in a TECAN infinite 200 (Durham, NC) plate reader, and MUC5AC was expressed as ng/mg tear protein.

### Statistical analysis

The sample size was calculated using StatMate 2 (GraphPad Software Inc., San Diego, CA, USA) based on pilot studies to have at least 80% power to detect a 20% difference between regions, with an alpha of 0.05. Demographic data was compared by ANOVA with Tukey post-hoc multiple comparisons. Based on Shapiro-Wilk normality testing, parametric student T (goblet cell count) or non-parametric Mann-Whitney U (goblet cell area) tests were performed for statistical comparisons with an alpha of 0.05 using GraphPad Prism 7.0 software (GraphPad Software Inc.). Pearson correlations between regional goblet cell numbers, severity markers (corneal fluorescein and conjunctival lissamine green staining), and tear MUC5AC concentration were measured.

## Results

Demographic and severity data are provided in Table 1. Both ATD groups had significantly lower TMH and greater corneal fluorescein and conjunctival lissamine green staining than the control group. SS ATD had greater ocular surface disease than non-SS ATD.

**Table 1:** Demographic characteristics of study groups.

Groups	Number	Age mean ± SD	Age range	TMH (μm)	K FL (0–15)	CJ LG (0–6)
Control	12	34.0 ± 12.84	25–64	310 ± 70	0	0
Non-SS ATD	9	53.89 ± 18.14	25–79*	193 ± 40.2*	3.2 ± 1.9*	2.1 ± 1.1*
SS ATD	7	55.13 ± 16.44	37–77*	113 ± 39*†	8.9 ± 3.9*†	4.5 ± 2.6*†
All ATD	16	54.47 ± 16.63	25–79*	162 ± 58	5.5 ± 3.7	3.1 ± 2.1

ATD = aqueous tear deficiency; SS = Sjögren syndrome; SD = standard deviation; TMH = tear meniscus height; K FL = corneal fluorescein staining score; Cj LG = conjunctival lissamine green staining score

*P < 0.05 compared to control; ^†^P < 0.05 compared to non-SS ATD.

### Comparison of goblet cell number and area in control and ATD

The goblet cell number and area in the TB and SB sites were compared between the ATD and control groups (Table 2). Goblet number and area in the TB conjunctiva were significantly lower than control in all the ATD groups. In the SB conjunctiva, goblet number was significantly lower than control only in the SS group, while SB goblet cell area was lower in the SS and all ATD groups. There were no differences in goblet cell parameters at either site between the SS and non-SS ATD groups.

**Table 2:** Goblet cell number and area in the TB and SB sites in ATD and control groups.

Parameter	Control	Non-SS ATD ± SD	SS ATD ± SD	All ATD ± SD
SB Count	221.7	173.9	87.16	134
	±126.7	±281.5	±75.7	±209.9
	CV = 56.9		P = 0.02	CV = 157.3
TB Count	310.5	75.56	89.52	82.08
	±254.2	±49.6	±78.2	±62.5
	CV = 81.8	P = 0.02	P = 0.04	P = 0.0004
				CV = 76.1
Total Count	252.6	120.1	88.30	106.2
	±157.1	±146.2	±72.9	±117.4
			P = 0.02	P = 0.009
SB Area	291.7 × 10^3^	201.0 × 10^3^	102.5 × 10^3^	155.0 × 10^3^
	±138.1 × 10^3^	±318.9× 10^3^	±97.7 × 10^3^	±239.9 × 10^3^
	CV = 47.3		P = 0.008	P = 0.05
				CV = 154.7
TB Area	517.1 × 10^3^	99.9 × 10^3^	96.1 × 10^3^	98.1 × 10^3^
	±403.8 × 10^3^	±80.3 × 10^3^	±56.8 × 10^3^	±67.9 × 10^3^
	CV = 78.1	P = 0.01	P = 0.02	P < 0.001
				CV = 69.2
Total Area	372.3 × 10^3^	134.8 × 10^3^	99.3 × 10^3^	119.2 × 10^3^
	±241.6 × 10^3^	±179.4 × 10^3^	±68.1 × 10^3^	±139.1 × 10^3^
		P = 0.03	P = 0.01	P = 0.002

P = ATD vs. control; ATD = aqueous tear deficiency; SS = Sjögren syndrome; SD = standard deviation; TB = temporal bulbar conjunctiva; SB = superior bulbar conjunctiva; CV = coefficient of variation.

### Regional comparison of goblet cell number and area within groups

Goblet cell number and area in the SB and TB regions were significantly correlated (P < 0.05) in all the ATD groups (non-SS, SS and all ATD) and in all subjects (r > 0.7 for number and area for all groups). There was no significant difference in goblet cell number or area between TB and SB in any group or in all subjects. The coefficient of variation (CV) for goblet cell number and area was greater for the SB than the TB sites in the ATD groups (Number CV = 157.3, CV = 76.1, respectively; Area CV = 154.7, CV = 69.2, respectively) and lower in the control group (Number CV = 56.9, CV = 81.8, respectively; Area CV = 47.3, CV = 78.1, respectively) (Table 2).

### Tear MUC5AC protein and correlations with goblet cell and clinical parameters

**Figure 2:**
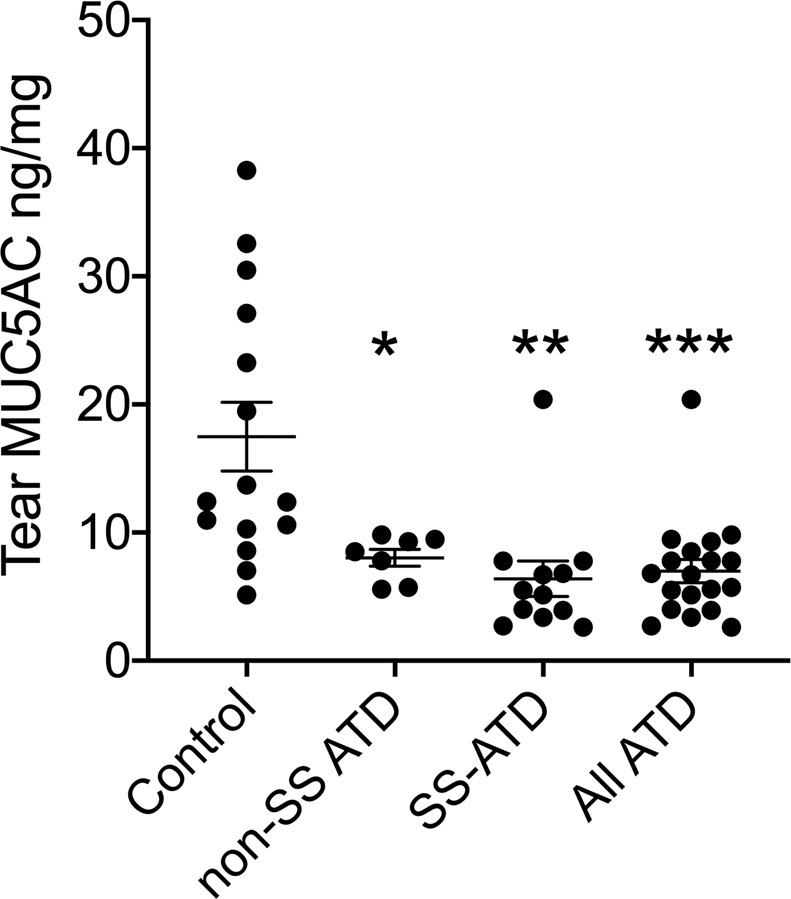
Tear MUC5AC concentration measured by ELISA in control and Sjögren syndrome (SS), non-SS associated and all aqueous deficient tear deficient (ATD) eyes. *P = 0.03; **P = 0.002; ***P = 0.0003 vs. control.

Normalized tear MUC5AC protein was significantly decreased in all ATD groups compared to the control group (Fig. 2). Tear MUC5AC was significantly correlated with goblet cell area (r = 0.48, P < 0.05 all subjects), but not with goblet cell number. Corneal fluorescein staining was significantly correlated with goblet cell area (r = −0.24, P = 0.04) and tear MUC5AC level (r = −0.56, P = 0.01), while conjunctival lissamine green staining was correlated with goblet cell count (r = −0.4, P = 0.04) and area (r = −0.53, P = 0.01), as well as tear MUC5AC (r = −0.59, P = 0.008) (Table 3).

**Table 3:** Correlations between ocular surface dye staining and goblet cell parameters and tear MUC5AC.

	GC Count	GC Area	Tear MUC5AC
K FL	NS	r = 0.24	r = 0.56
		p = 0.04	p = 0.01
Cj LG	r = 0.40	r = 0.53	r = 0.59
	p = 0.04	p = 0.01	p = 0.008

K FL = corneal fluorescein staining score; Cj LG = conjunctival lissamine green staining score; NS = not statistically significant; r = Pearson correlation coefficient.

## Discussion

Our study compared regional goblet cell number in the exposed TB and covered SB conjunctiva in control eyes to those of non-SS and SS-associated ATD. Because of the observed variation in goblet cell size, we also measured goblet cell area using image analysis software, which to our knowledge has not been previously described. Similar to previously reported studies, we found decreased goblet cell number in the exposed TB conjunctiva in both ATD groups^4–7^. Goblet cell number in the SB was also lower than control eyes in the SS and all ATD groups. Goblet cell area was also significantly lower than control in the TB and SB regions in SS and all ATD, but only in the TB in the non-SS group. The more extensive goblet cell loss in SS may be due to greater levels of inflammatory mediators in the conjunctiva and tears or to the lower tear volume in SS than non-SS ATD^23–25^.

While measuring goblet cell number in this and previously reported studies from our group, we have noted marked variability in goblet size, particularly in dry eye samples where some cells may be very small. In mouse models of ATD, small or poorly filled goblet cells have been noted to have signs of an unfolded protein response or apoptosis^11^. For this reason, we also evaluated goblet cell area to determine if it shows greater discrimination between groups than goblet cell number. Among all subjects with ATD, goblet cell number was significantly lower than control in the TB, but not SB; however, goblet cell area was significantly lower than control in both regions. This suggests that goblet cell area may be a better measure of goblet cell disease and dysfunction than goblet cell number in ATD; however, this requires further validation.

Age is a known risk factor for dry eye^26–28^ and conjunctival goblet cell loss^29,30^. As such, finding age-matched control subjects is difficult because the prevalence of dry eye and conjunctival goblet cell loss increases with age. Consequently, our control group is younger than the dry eye groups.

### Variation in goblet cell parameters between the SB and TB conjunctiva

There are conflicting results regarding regional difference in goblet cell number in the SB and TB conjunctiva in previously reported studies. A meta-analysis of 33 studies across 25 years by Doughty described higher mean goblet cell number in the covered conjunctiva, including the SB region (973 +/− 789 cells/mm^2^), compared to the exposed TB conjunctiva (427 +/−376 cells/mm^2^)^31^. However; this review calculated large standard deviations within groups which may be a factor contributing to the wide variation in reported goblet cell densities from study to study. Although goblet cell number in the SB was numerically decreased compared to TB in control eyes in our study, we found no statistically significant differences between SB and TB densities, and both goblet number and area in these two sites were significantly correlated in ATD and all samples. We also calculated a large standard deviation and higher coefficient of variation in SB goblet cell parameters in all groups, despite strict control and standardization techniques. We did not find any association between low SB goblet cell number or area and greater cornea or conjunctival dye staining. None of the ATD subjects had clinical signs of superior limbic keratoconjunctivitis, a condition where greater reduction of goblet cell number has been observed in the SB vs. TB conjunctival sites. Komuro *et al*. reported consistently lower goblet cell number in the SB conjunctiva in eyes with SLK (11/11 samples) than SS-associated ATD (3/13 samples) (p = 0.004)^32^. These results are consistent with our findings of no significant difference in goblet cell parameters between the TB and SB sites in eyes with non-SS or SS ATD.

### Tear MUC5AC protein may be a surrogate marker for goblet cell area

Tear MUC5AC protein was lower than control in all ATD groups and correlated with goblet cell area. Additionally, tear MUC5AC concentration correlated with severity of corneal fluorescein and conjunctival lissamine green staining. Uchino *et al*. previously reported that tear MUC5AC concentration was significantly correlated with goblet cell density in office workers^33^. Taken together, these findings suggest that tear MUC5AC is a disease-relevant biomarker for conjunctival goblet cells. Once a standardized method is developed, it could be used for diagnostic classification and grading severity of and efficacy of therapies for conjunctival disease in ATD.

In conclusion, goblet cells were significantly decreased in covered and exposed conjunctiva in SS eyes compared to control eyes in our study. Although goblet cell number was lower in the SB than the TB in control eyes, this was not statistically significant. Due to variability in goblet cell size, our study suggests that goblet cell area may be a better discriminatory measure of goblet cell disease and dysfunction than goblet cell number in ATD, though this requires validation in future studies. Correlation between tear MUC5AC concentration and GC area suggests tear MUC5AC mucin can be used as a disease-relevant biomarker for conjunctiva GC health once a standardized methodology is developed.
